# Factors associated with suicidal attempts in female patients with mood disorder

**DOI:** 10.3389/fpubh.2023.1157606

**Published:** 2023-09-25

**Authors:** Jinhe Zhang, Sixiang Liang, Xinyu Liu, Dan Li, Fuchun Zhou, Le Xiao, Jun Liu, Sha Sha

**Affiliations:** ^1^Beijing Key Laboratory of Mental Disorders, The National Clinical Research Center for Mental Disorders, The Advanced Innovation Center for Human Brain Protection, Beijing Anding Hospital, Capital Medical University, Beijing, China; ^2^The Advanced Innovation Center for Human Brain Protection, Capital Medical University, Beijing, China

**Keywords:** mood disorder, prediction model, suicidal attempt, Chinese population, LASSO Logistic Regression

## Abstract

**Aim:**

This study aims to establish a nomogram model to predict the relevance of SA in Chinese female patients with mood disorder (MD).

**Method:**

The study included 396 female participants who were diagnosed with MD Diagnostic Group (F30–F39) according to the 10th Edition of Disease and Related Health Problems (ICD-10). Assessing the differences of demographic information and clinical characteristics between the two groups. LASSO Logistic Regression Analyses was used to identify the risk factors of SA. A nomogram was further used to construct a prediction model. Bootstrap re-sampling was used to internally validate the final model. The Receiver Operating Characteristic (ROC) curve and C-index was also used to evaluate the accuracy of the prediction model.

**Result:**

LASSO regression analysis showed that five factors led to the occurrence of suicidality, including BMI (*β* = −0.02, SE = 0.02), social dysfunction (*β* = 1.72, SE = 0.24), time interval between first onset and first dose (*β* = 0.03, SE = 0.01), polarity at onset (*β* = −1.13, SE = 0.25), and times of hospitalization (*β* = −0.11, SE = 0.06). We assessed the ability of the nomogram model to recognize suicidality, with good results (AUC = 0.76, 95% CI: 0.71–0.80). Indicating that the nomogram had a good consistency (C-index: 0.756, 95% CI: 0.750–0.758). The C-index of bootstrap resampling with 100 replicates for internal validation was 0.740, which further demonstrated the excellent calibration of predicted and observed risks.

**Conclusion:**

Five factors, namely BMI, social dysfunction, time interval between first onset and first dose, polarity at onset, and times of hospitalization, were found to be significantly associated with the development of suicidality in patients with MD. By incorporating these factors into a nomogram model, we can accurately predict the risk of suicide in MD patients. It is crucial to closely monitor clinical factors from the beginning and throughout the course of MD in order to prevent suicide attempts.

## 1. Introduction

Suicide is defined as intentional self-directed harm resulting in death. According to the World Health Organization (WHO) statistics, approximately 70.3 million people commit suicide each year, equating to one suicide every 40 s (1). Suicide can occur at various stages of the life cycle. A global study conducted in 2008 revealed that the lifetime prevalence of suicidal ideation and attempts is 9.2 and 2.7%, respectively. Furthermore, one-third of individuals with suicidal thoughts make a suicide plan, and another third attempt suicide (2). Since 2019, suicide has ranked as the fourth leading cause of death among individuals aged 15–29 years worldwide (3).

In recent years, numerous studies have investigated the mechanisms underlying suicide to identify accurate risk factors. Previous research has suggested that Hypothalamic–pituitary–adrenal (HPA) axis activity is implicated in suicide risk, regardless of whether psychiatric conditions are present or not. Additionally, abnormalities in the HPA axis, particularly hyperactivity, may significantly influence suicide risk, while impaired stress response mechanisms also contribute to this risk (4). Moreover, individuals at risk for suicide have been found to exhibit higher mean concentrations of inflammatory mediators in both the peripheral and brain tissues (5). However, these inflammatory and hormonal risk factors are not entirely reliable predictors of suicide in patients with mental disorders, and their clinical utility is limited.

Conversely, many researchers propose that approximately 90% of suicide attempts are accompanied by a mental disorder, with emotional disorders being one of the primary contributing factors (6). The situation is particularly severe among patients with mood disorders (MD), as they have a significantly higher risk of suicidal death, with a prevalence 20–30 times greater than that of the general population (7). The standardized death rate (SMR) from suicide among individuals with unipolar depression is more than 20 times higher than that of the general population (8). Additionally, suicide accounts for approximately 15% of deaths related to bipolar disorder (BD) (9). These findings highlight the urgent need for improved suicide prevention and treatment. Furthermore, controlling emotional disorders has been shown to reduce the incidence of serious suicide attempts (SA) by 80% (6). Risk factors for suicide have been explored using machine learning, decision tree analysis, and neural networks (10–12). However, most studies have examined both males and females together, with limited research specifically addressing suicide risk factors in women with MD. One study found that suicide attempts in individuals with affective disorders are more common among females (13). Additionally, it revealed that girls aged 12 to 19 have a higher incidence of suicide attempts compared to boys (14). De Leo’s study indicated that nearly 90% of individuals who died by suicide had interacted with some form of healthcare professional within 3 months prior to their death (15). In clinical practice, it is crucial to identify risk factors associated with suicide attempts in patients with MD for early detection and risk reduction. While there is a substantial amount of literature on the risk and prevention of suicide attempts, limited research has focused specifically on identifying these risk factors. Additionally, there is currently no predictive model based on social demographic information that can be used in clinical practice to predict female suicide attempts.

This study aims to identify effective predictors for suicide risk in female patients with MD who have attempted suicide by analyzing their sociodemographic and clinical characteristics. The goal is to develop a predictive model that establishes the association between suicide attempts and diagnosed affective disorders in females. To achieve this, the study will utilize the LASSO Logistic Regression model and nomogram model to systematically analyze risk factors related to suicide attempts in MD patients and establish a reliable predictive model.

## 2. Methods

### 2.1. Study design

The present study was conducted at Beijing Anding Hospital from 2019 to 2021 and designed as a cross-sectional retrospective analysis. Two attending psychiatrists independently enrolled patients based on the diagnostic criteria for mood disorders in the International Statistical Classification of Diseases and Related Health Problems 10th edition (ICD-10) (16). All participants were informed about the research at the time of admission and provided consent for the anonymous sharing of their medical records. The study was reviewed and approved by the Ethics Committee of Beijing Anding Hospital.

### 2.2. Inclusion and exclusion criteria

The complete electronic medical records of female patients who met the admission criteria were transferred and reviewed by specialized psychiatrists. Personal information was removed to ensure anonymity. Patients with other psychiatric disorders such as schizophrenia, schizoaffective disorder, personality disorder, and intellectual disability were excluded from the study. Patients with a history of psychiatric illness comorbid with alcohol or drug abuse were also excluded. The use of atypical antipsychotics or antidepressant medications did not influence the data collection of enrolled patients.

### 2.3. Basic characteristics and definition

Detailed sociodemographic profiles and clinical characteristics were collected, including age, education, employment status, body mass index (BMI), marital status, income, residential address, age of first onset, duration of first onset, medication time (since first dose), psychotic symptoms at the first episode, polarity at onset, duration of overall illness, times of hospitalizations, social dysfunction, family history of mood disorders, and somatic diseases.

Social function refers to a person’s ability to manage everyday activities, engage in leisure and occupational activities (17). The Personal Social Performance (PSP) scale was used to assess functioning across four dimensions, including socially useful activities, personal and social relationships, self-care, and disturbing and aggressive behaviors (18). Patients were categorized into two groups, with or without social dysfunction, based on a cutoff score of 70.

Following Silverman et al. (19), suicidal attempts (SA) included suicidal thoughts (ST) and suicide plans (SP). Suicide assessment was conducted by specialized psychiatrists who assessed and interviewed the patients during episodes. Based on the patients’ affirmative responses to questions such as “Have you ever thought about suicide?” “Have you ever had a plan for how to kill yourself?” and “Have you ever tried to kill yourself?” all participants were classified into two groups: mood disorder patients with suicidal attempts (MD-S) and mood disorder patients without suicidal attempts (MD-N). These questions are commonly used in suicidality studies worldwide (20, 21). All evaluation records were stored in the electronic medical record system.

### 2.4. Statistical analysis

Statistical analyses were performed using STATA software (version 14.0, Stata Corp, TX) and the R programming environment (version 4.0.2). Categorical data were presented as counts (percentages), while continuous data were presented as medians (interquartile ranges). Two-group comparisons were conducted using the Wilcoxon rank-sum test or χ2 test, as appropriate. A two-sided *p*-value of less than 5% was considered statistically significant for comparisons between MD-S and MD-N. Multiple imputations were performed using the R MI package with five replications and a chained equations approach to handle missing data (22).

The LASSO regression model (23) was used to identify the risk factors associated with suicidal attempts in MD patients by quantifying the contribution of all possible factors and selecting relevant predictors while avoiding overfitting. The logistic LASSO regression was fitted using the “glmnet” package (version 2.0–16), and the penalty term was chosen using tenfold cross-validation. The selected factors of statistical significance formed the elements of the prediction model. A predictive nomogram model was created using the R “rms” package to facilitate the early identification of suicidal behavior in MD patients. The discrimination ability of the nomogram model was evaluated using the area under the receiver-operator characteristic (ROC) curve (AUC). The predictive accuracy of the nomogram model was assessed using calibration plots and the C-index. A C-index >0.7 indicates a well-fitted predictive model. Internal validation of the nomogram model was performed using bootstrap resampling with 100 replications (24, 25). This approach allows for the application of internally validated samples through repeated sampling, and the average value is calculated from 100 repetitions to determine the C-statistics after optimistic adjustment.

## 3. Results

### 3.1. Demographic characteristics

A total of 396 hospitalized female patients (mean age = 35.81 years, SD =15.65) were included in the present study, and 48% of the patients had suicidal attempt. The baseline characteristics of all the participants are shown in Table 1. In the comparison between MD-S and MD-N, there were significant differences in patients’ age, duration of overall illness, age of first onset, times of hospitalizations, polarity at onset, and social dysfunction. Contrary, we found no statistical differences between these two groups in terms of education, employment status, BMI, marital status, income, residential address, duration of first onset, psychotic symptom(s) at first episode, family history of mental disorders, and somatic diseases.

**Table 1:** Demographic information and clinical characteristics of study participants in this study.

Characteristics	MD-N (*n* = 206)	MD-S (*n* = 190)	*p*
Age (years)	35.5 (25.0, 49.0)	31.0 (20.0, 48.0)	0.028
BMI (kg/m^2^)	23.6 (20.6, 27.1)	22.9 (19.7, 27.1)	0.207
Education (years)	13.0 (12.0, 16.0)	13.0 (12.0, 15.0)	0.841
Marital status (*n*, %)			0.451
Unmarried	114 (55.3%)	115 (60.5%)	
Widowhood or divorce	20 (9.7%)	13 (6.8%)	
Married	72 (35%)	62 (32.6%)	
Job (*n*, %)			0.380
Manual labour	9 (4.4%)	9 (4.7%)	
Mental labour	67 (32.5%)	74 (38.9%)	
Unemployed	130 (63.1%)	107 (56.3%)	
Family history (*n*, %)			0.605
No	148 (71.8%)	132 (69.5%)	
Yes	58 (28.2%)	58 (30.5%)	
Family income (*n*, %)			0.610
<¥1,000	27 (13.1%)	32 (16.8%)	
¥1,000–3,000	61 (29.6%)	47 (24.7%)	
¥3,000–5,000	54 (26.2%)	50 (26.3%)	
>¥5,000	64 (31.1%)	61 (32.1%)	
Residential address (*n*, %)			0.442
Cities	149 (72.3%)	148 (77.9%)	
Villages and towns	12 (5.8%)	9 (4.7%)	
Countryside	45 (21.8%)	33 (17.4%)	
Duration of overall illness (months)	72 (25, 143)	48 (12, 120)	0.011
Age of onset (years)	24.5 (18.0, 35.0)	20.5 (16, 33)	0.043
Duration of first-onset (months)	3.67 (5.14)	3.96 (4.66)	0.128
Medication time	5.47 (8.52)	8.12 (14.82)	0.142
Times of hospitalizations	1.80 (1.92)	1.98 (1.99)	0.001
Polarity at onset			
Depression	149 (72.3%)	171 (90%)	<0.001
Mania	42 (20.4%)	15 (7.9%)	
Paranoia	15 (7.3%)	4 (2.1%)	
Social dysfunction			<0.001
No	122 (59.2%)	54 (28.4)	
Yes	84 (40.8%)	136 (71.6%)	
Psychiatric symptoms of first-onset			0.996
No	141 (68.4%)	130 (68.4%)	
Yes	65 (31.6%)	60 (31.6%)	
Somatic diseases			0.869
No	8 (3.9%)	8 (4.2%)	
Yes	198 (96.1%)	182 (95.8%)	
BMI (kg/m^2^)			0.253
<25	123 (59.7%)	122 (64.2%)	
25–30	52 (25.2%)	35 (18.4%)	
>30	31 (15%)	33 (17.4%)	

BMI, body mass index. Data are expressed as median (interquartile range) or count (percent). *p*-value was calculated by the rank-sum test or the χ ^2^ test, where appropriate.

### 3.2. Identification of potential factors

**Figure 1:**
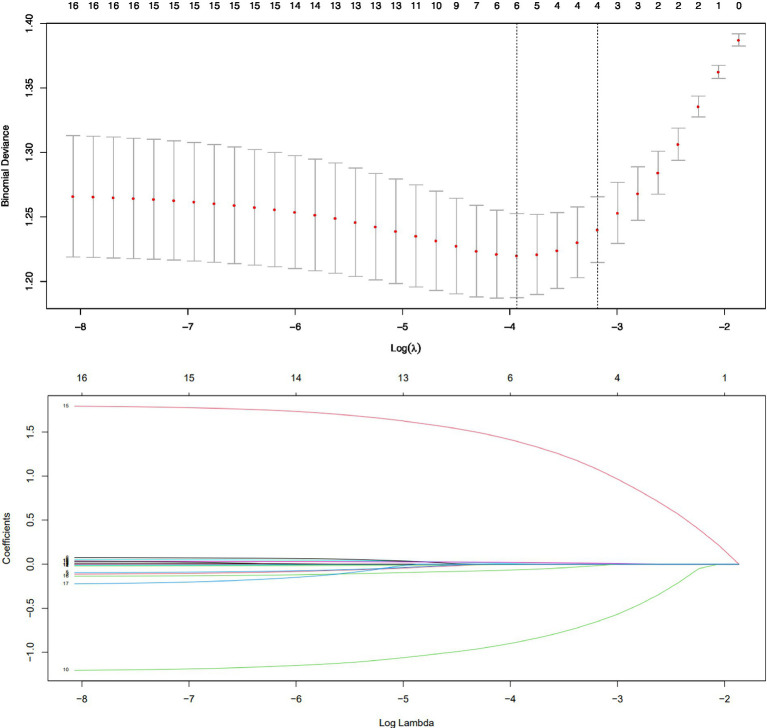
Cross validation plot for the penalty term and plots for LASSO regression over different values of the penalty parameter.

Totally 18 variables were included in the LASSO regression. Table 2 shows the estimated coefficients of the candidate factors for SA among MD patients. For physician-diagnosed SA, the λ values in this study ranged from 0.000313 to 0.154700, with an optimal λ of 0.023620 (Figure 1). Finally, LASSO regression analysis showed that five factors led to the occurrence of suicidality, including BMI (*β* = −0.02, SE = 0.02), social dysfunction (*β* = 1.72, SE = 0.24), time interval between first onset and first dose (*β* = 0.03, SE = 0.01), polarity at onset (*β* = −1.13, SE = 0.25), and times of hospitalization (*β* = −0.11, SE = 0.06).

**Table 2:** The estimated coefficients for logistic least absolute shrinkage and selection operator (LASSO) regression between candidate risk factors with SA.

Variables	Coefficients (SE)
BMI	−0.02 (0.02)
Social dysfunction	1.72 (0.24)
Time interval between first onset and first dose	0.03 (0.01)
Polarity at onset	−1.13 (0.25)
Times of hospitalization	−0.11 (0.06)

### 3.3. Risk prediction nomogram models

**Figure 2:**
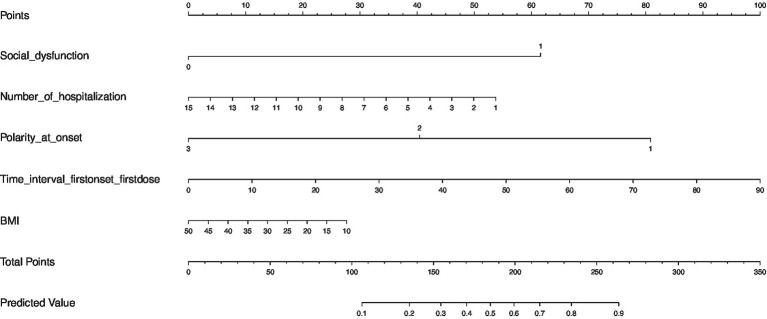
The prediction nomogram of risk factors for SA in female patients with mood disorder.

Since the relative contribution of each individual factor to the development of SA in MD may be small, it is important to consider the combined contribution of selected significant factors. We constructed a risk prediction nomogram model for SA in MD patients with the combination of the 5 risk factors mentioned above (Figure 2). To better understand the utility of the nomogram model, an example is given here. The probability of SA in a MD patient was estimated to be 75% assuming that the patient had social impairment (64 points), had 1 time of hospitalization (55 points), had a diagnosis of depression at the first onset (82 points), had an interval of 30 months from the first onset to the first dose (34 points), and had a BMI of 35 (10 points).

**Figure 3:**
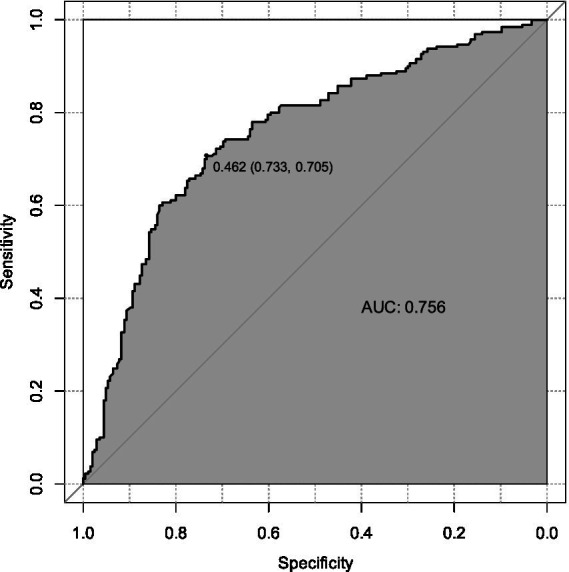
The ROC curve between candidate risk factors with SA.

**Figure 4:**
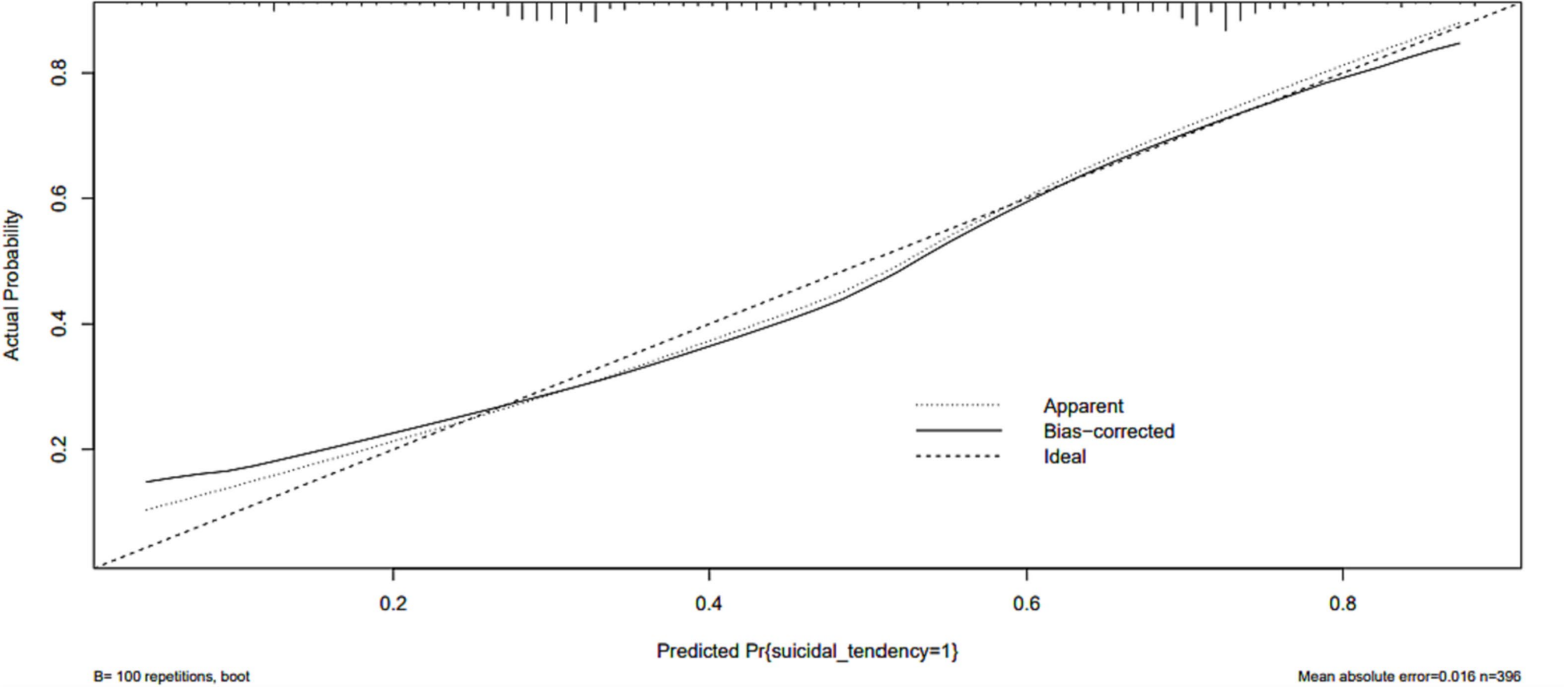
The logistic calibration curve of the prediction nomograms of risk factors for SA in female patients with mood disorder.

The ROC analysis assessed the ability of the nomogram model to recognize suicidality, with good results (AUC = 0.76, 95% CI: 0.71–0.80) (Figure 3). The predictive accuracy of the nomogram was determined using a calibration plot (Figure 4) with a C-index of 0.756 (95% CI, 0.750–0.758), which indicated that the model had good discrimination and predictive accuracy.

### 3.4. Internal validation

The constructed nomogram model was internally validated by Bootstrap resampling with 100 replicates. For MD with SA, the C-statistic in the derived cohort was 0.756 with good internal validation (bootstrapped C-statistic of 0.740) and excellent calibration of predicted and observed risks.

## 4. Discussion

In this study, we found five factors including BMI, social dysfunction, time interval between first onset and first dose, polarity at onset, and times of hospitalization were significantly associated with the development of suicidality in MD patients. Specifically, we found that individuals who experienced depression at onset (as opposed to manic episodes) and those with social dysfunction were more likely to attempt suicide. On the other hand, higher BMI, timely hospitalization, and shorter intervals between first onset and medication initiation were associated with a lower risk of suicidal attempts. Importantly, the inclusion of these factors in the nomogram model can strongly predict the risk of suicide in MD patients with considerable accuracy.

In detail, we concluded that MD patients with a first depressive episode was associated with a higher risk of SA. A prospective study by Daban et al. (26) involving 300 patients with BD with a 10 years follow-up showed that patients with depressive episodes had a higher rate of SA. Results from another study in Barcelona (27), which recruited 224 patients with BD, also showed that lifetime history of suicide attempts was strongly associated with depression polarity and that the average number of SA was higher in patients with depression polarity.

Generally, suicide risk varied according to illness characteristics and stage of illness, with suicidal behavior mainly associated with depression and mixed phase illness. Major depressive episodes were associated with the highest risk of suicide, followed by the mixed phase and finally the manic phase, which was associated with the lowest risk of suicide (7). SA were more common during the first depressive episode and at the early onset of the illness, rather than later in the illness (28, 29). Thus, our study supports previous conclusions that first-episode depression with SA needs more clinical attention.

Our LASSO Logistic Regression analysis suggests that individuals with mood disorders (MD) and increased BMI are less likely to exhibit suicidal tendencies. These findings may be attributed to the treatment of MD, as it is well-recognized that regular use of drugs for psychiatric disorders can lead to adverse effects such as weight gain. For instance, antipsychotics (AP) used in the treatment of schizophrenia are known to cause weight gain in a significant percentage of patients, ranging from 15 to 72% (30–34). Similarly, chronic users of antidepressants often experience adverse effects of weight gain, with 65.3% of patients reporting such effects (35, 36). Clozapine and olanzapine are reported to have the greatest risk of weight gain, while quetiapine and risperidone pose an intermediate risk (37–39). Given that these medications are commonly used in the treatment of mood disorders, our results suggest that better medication adherence may reduce the risk of suicidal ideation in patients.

Regarding social dysfunction, our findings highlight that it is a risk factor that exacerbates suicidal behavior in individuals with MD. Appropriate social functioning forms the basis for the formation and development of satisfying and lasting relationships, which are essential for physical and psychological well-being throughout the lifespan (40). However, patients with MD are prone to experiencing social dysfunction, which has been consistently linked to poor health outcomes and premature death for decades (41, 42). Studies have demonstrated an association between social dysfunction and suicidal attempts in individuals with MD (43), aligning with the conclusions we have provided. Social dysfunction manifests in various aspects of socialization, including difficulties in dealing with social stress and problems, reduced ability to recognize emotions in others, poor social integration, and impaired social support (44–46). These factors have been confirmed to be related to self-harm, suicidal thoughts, and suicide attempts. MD itself exacerbates social cognitive deficits in patients, which can further impact the progression of the disease. Therefore, comprehensive treatment for bipolar disorder requires not only medication but also social cognitive interventions to achieve comprehensive psychological stability (47).

The results of our analysis showed that two risk factors, the number of hospitalizations in MD patients and the interval between the first onset of first dose, had negative and positive feedback, respectively, with suicidal tendency. On the one hand, the former results of our analysis mean that patients who actively receive inpatient treatment with cooperative doctors have relatively stable disease control. On the other hand, if the first depressive or manic episode requires timely medication, the delay in the condition causes a poor outcome. However, study demonstrated that delays in help-seeking were common in MD patients even in offspring of patients with depressive and/or anxiety disorders (48). This information may help to develop targeted strategies to reduce help-seeking delays.

This study has several limitations. First, the nature of this study was cross-sectional and could not confirm the causal relationship between risk factors and outcomes, because the time dimensions of risk factors and SA do not correspond (with some “risk factors” occurring after SA onset). Although an advanced analytic technique is employed, the data are cross-sectional, risk factors studied in this manuscript are well-established that make interpretation of results attenuated. So future cohort studies with higher levels of evidence are needed for further determination. Secondly, the lack of external validation prevents the nomogram model from being better replicated in other centers. However, the results of our single-center study may provide a reference for suicide prevention for female with MD nationwide. Thirdly, further sample size expansion is needed. An insufficient sample size might have influenced the validity of the nomogram model.

## 5. Conclusion

In summary, several factors influence the development of suicidality in individuals with mood disorders, including polarity of onset, social dysfunction, BMI, number of hospitalizations, and interval between first onset and medication. The nomogram model developed in this study holds potential in facilitating early identification and implementing prevention strategies, thereby improving the prognosis of suicidal attempts in this population.

## Data availability statement

The original contributions presented in the study are included in the article/supplementary material, further inquiries can be directed to the corresponding author.

## Ethics statement

The studies involving humans were approved by Beijing Anding Hospital, Capital Medical University. The studies were conducted in accordance with the local legislation and institutional requirements. Written informed consent for participation was not required from the participants or the participants' legal guardians/next of kin because the present study was designed as a cross-sectional retrospective analysis. All participants were informed about the research at the time of admission and provided consent for the anonymous sharing of their medical records.

## Author contributions

SS had full access to all the data in the study and takes responsibility for the integrity of the data and the accuracy of the data analysis. JZ and SL: formal analysis, methodology, writing – original draft, and writing – review and editing. XL, DL, FZ, and LX: data curation and conceptualization. JL: critical revision of the manuscript for important intellectual content. All authors contributed to the article and approved the submitted version.

## Funding

This study was supported by High-level Public Health Technology Talent Development Program, Project Training Plan Number: Discipline Backbone-02-37.

## Conflict of interest

The authors declare that the research was conducted in the absence of any commercial or financial relationships that could be construed as a potential conflict of interest.

## Publisher’s note

All claims expressed in this article are solely those of the authors and do not necessarily represent those of their affiliated organizations, or those of the publisher, the editors and the reviewers. Any product that may be evaluated in this article, or claim that may be made by its manufacturer, is not guaranteed or endorsed by the publisher.
